# Implementing HPV self-collection: a scoping review of facilitators and strategies among Indigenous women and people with a cervix

**DOI:** 10.1186/s12889-026-26720-x

**Published:** 2026-02-23

**Authors:** Louise Mitchell, Deb Wong, Tamara Butler, Julia Brotherton, Joan Cunningham, Sonya Egert, Kristine Falzon, Gail Garvey, Beverley Lawton, Claire Nightingale, Marion Saville, Natalie Taylor, Claudette Tyson, Kate Wilkinson, Claire Zammit, Lisa J. Whop

**Affiliations:** 1 Yardhura Walani, National Centre for Aboriginal and Torres Strait Islander Wellbeing Research, Australian National University, Canberra, Australian Capital Territory Australia; 2 First Nations Cancer & Wellbeing Research Program, The School of Public Health, Faculty of Medicine, The University of Queensland, Brisbane, Queensland Australia; 3 Evaluation and Implementation Science Unit Centre for Health Policy, Melbourne School of Population & Global Health, University of Melbourne, Victoria, Australia; 4 Menzies School of Health Research, Charles Darwin University, Darwin, NT Australia; 5 Inala Wangarra, Inala, Queensland Australia; 6 Waminda South Coast Women’s Health and Wellbeing Aboriginal Corporation, Nowra, New South Wales Australia; 7 Te Tātai Hauora o Hine-National Centre for Women’s Health Research Aotearoa, Faculty of Health, Victoria University of Wellington, Wellington, Aotearoa New Zealand; 8 Centre for Health Policy, Melbourne School of Population and Global Health, University of Melbourne, Melbourne, Australia; 9 Australian Centre for Prevention of Cervical Cancer, Melbourne, Australia; 10 School of Population Health, Faculty of Medicine and Health, UNSW Sydney, High Street Kensington, Sydney, NSW 2052 Australia; 11 Queensland Centre of Excellence in Aboriginal and Torres Strait Islander Primary Health Care, Inala, Queensland Australia

**Keywords:** Cervical screening, Cancer prevention, Self-collection, Human Papillomavirus, Scoping review, Indigenous peoples, Implementation, Strategies, Barriers, Facilitators

## Abstract

**Background:**

Indigenous peoples in high-income settler-colonial countries experience disproportionately high cervical cancer incidence and mortality compared to non-Indigenous populations, reflecting systemic inequities and barriers to culturally safe care. HPV self-collection offers a potential solution to overcoming inequities by enabling women and people with a cervix to collect their own sample, improving autonomy, privacy, and access, and supporting culturally responsive models of care. While self-collection is accurate, acceptable, and increases screening participation, evidence is needed on how best to support implementation to reach Indigenous women and people with a cervix.

**Objectives:**

This scoping review aimed to identify and describe implementation strategies, barriers, facilitators, and context-specific adaptations to HPV self-collection for cervical screening among Indigenous women and people with a cervix in high-income settler colonial countries.

**Methods:**

Eligible sources included peer-reviewed and grey literature published between August 2013 and November 2025 that described barriers, facilitators, implementation strategies, or context-specific adaptations to HPV self-collection among Indigenous women and people with a cervix in Australia, Aotearoa New Zealand, Canada, or the United States of America (USA). Database (PubMed, SCOPUS, ProQuest CINAHL) and grey literature searches targeting Indigenous-specific organisations, government websites were conducted, using the broad search terms: cervical screening, HPV, self-collection, and Indigenous peoples. Data were extracted and mapped against the Health Equity Implementation Framework to identify barriers, facilitators, and strategies relevant to the implementation of HPV self-collection.

**Results:**

32 publications were included, comprising 28 academic publications and 4 from the grey literature. Identified facilitators included client health literacy and knowledge, flexible models of care, culturally responsive care, supportive data and systems, and strengths-based messaging. Indigenous-led solutions were central to enabling these facilitators.

**Conclusions:**

While evidence comparing the impact of individual strategies is not available, community-driven, multi-component strategies were recommended. The most effective strategies appeared to combine flexible delivery models, such as home-based or outreach approaches, with tailored education focusing on increasing knowledge and building trust. Indigenous-led approaches and the involvement of Indigenous health workers and community leaders were central to strategies.

**Supplementary Information:**

The online version contains supplementary material available at 10.1186/s12889-026-26720-x.

## Background

Cervical cancer can be eliminated as a public health problem through the scale up of Human Papillomavirus (HPV) vaccination, primary HPV cervical screening programs and treatment of early-stage disease [1]. Australia is “on track” to become the first country to actively eliminate cervical cancer [2]. However, elimination is unlikely to be achieved among priority populations who carry a greater burden of disease, such as Aboriginal and Torres Strait Islander people where cervical cancer incidence is 11.7 per 100,000 women (2017–2021) and mortality is 4.3 per 100,000 (2019–2023; [3]. These rates are approximately two- and three- times more than the broader Australian population, respectively [3]. Similar inequities in cervical cancer are experienced by Indigenous peoples from other high-income settler-colonial countries, such as Aotearoa New Zealand, the United States of America (USA) and Canada (Turtle Island). The cervical cancer disease burden experienced by Indigenous women and people with a cervix remains unacceptably high when compared to non-Indigenous peoples of screening age [4]. These disparities highlight a screening system that fails to meet the needs of Indigenous peoples, shaped by systemic inequities and longstanding barriers to access, including culturally unsafe services and structural racism. To achieve elimination, cervical cancer incidence must be reduced by 66% among Aboriginal and Torres Strait Islander women and people with a cervix [3], and 63% among Māori women and people a cervix in Aotearoa New Zealand [4]. Data for Indigenous peoples in Canada and the USA were not published in sufficient detail to assess progress against elimination targets [4]. This lack of consistent and disaggregated data across countries limits the ability to fully understand and target efforts to achieving cervical cancer elimination.

Historically lower cervical screening participation rates among Indigenous peoples have driven efforts to improve screening access [4], including the introduction of HPV self-collection. Self-collection offers a potential solution to achieving the equitable elimination of cervical cancer, as it allows women and people with a cervix to take their own vaginal sample using a swab or other collection device which is then tested for HPV. Self-collection is as accurate as clinician collected samples, with meta-analysis showing comparable sensitivity for detecting cervical precancer (CIN2+) when using PCR-based assays [5]. Self-collection affords participants greater autonomy, privacy, comfort and convenience [6]. It also supports more flexible, culturally responsive delivery models to improve access for Indigenous women and people with a cervix [4, 7–11]. While available evidence finds HPV self-collection to be highly acceptable and capable of increasing cervical screening participation, in trial and organised program settings [9, 11–14], evidence is needed to support implementation approaches that are tailored to the distinct cultural contexts and priorities of Indigenous women and people with a cervix.

This implementation research is particularly important as more high-income settler-colonial countries introduce primary HPV testing within national screening programs, with self-collection as either an option or the primary method of collection [15, 16]. Australia and Aotearoa New Zealand have transitioned to HPV-based cervical screening, offering self-collection to all participants. Australia introduced HPV self-collection in 2017 for under-and never-screened screening participants, expanding access to all screen-eligible women and people with a cervix in 2022. Aotearoa New Zealand followed in 2023, adopting HPV primary screening with self-collection (referred to as “self-testing”) as the primary method with clinician-collected options available. Canada and the USA are progressing more incrementally. Within Canada, many jurisdictions are working towards adopting primary HPV testing with the option of self-collection [17], and in the USA, the American Cancer Society updated its guidelines in 2020 to recommend primary HPV screening as the preferred strategy, though implementation varies by state and provider [18]. These varied approaches reflect differences in health system structures and policy environments but collectively highlight recognition of self-collection’s potential to improve access and equity in cervical screening.

However, overcoming barriers to screening through the implementation of HPV self-collection alone is unlikely to achieve equity. Lower screening rates, partly due to invasive clinical examinations, contribute to inequities in cervical cancer outcomes among Indigenous peoples, however attributing these disparities solely to individual participation oversimplifies the issue. The introduction of HPV self-collection must be understood within the broader context of the enduring impacts of colonisation, including systemic racism, and structural barriers within healthcare systems which have led to reduced trust and engagement with screening programs [19]. These settler-colonial harms are ongoing. They are embedded in social systems and structures that maintain disparities and exclude Indigenous peoples from decision-making.

To ensure HPV self-collection supports equity, its implementation must be guided by approaches that acknowledge and respond to the unique needs and rights of Indigenous peoples. Further evidence is needed to understand how best to engage both practitioners and participants to optimise and support its implementation, particularly among under- and never-screened Indigenous women and people with a cervix.

## Methods

The authors are a team of Indigenous researchers (LJW, TB, SE, KF, GG, BL, CT) and non-Indigenous researchers (LM, DW, JB, JC, CN, MS, NT, KW, CZ) whose perspectives are shaped by their diverse cultural backgrounds and knowledge systems. Together, the team acknowledges that these positionalities influence how literature is interpreted and synthesised.

This scoping review aimed to identify barriers and facilitators of self-collection and implementation strategies in high-income settler-colonial countries and support equitable cervical cancer elimination efforts of cervical cancer among Indigenous peoples. In conducting this scoping review, we employed the stages identified by Arksey and O’Malley [20]. We applied the PRISMA-ScR (Preferred Reporting Items for Systematic Reviews and Meta-Analyses extension for Scoping Reviews) [21] to guide the search strategy, study selection, and reporting (see Supplemental File).

### Identifying the research question

The research questions for this review were: What strategies have been suggested or used to facilitate self-collection among Indigenous women and people with a cervix? What barriers or facilitators have these strategies addressed or intended to address? For clarification, the population of interest was Indigenous peoples eligible for cervical screening (i.e. both women and people with a cervix) from high-income settler-colonial countries, specifically Australia, Aotearoa New Zealand, the USA and Canada (Turtle Island).

While referred to collectively as Indigenous peoples, the authors respectfully acknowledge the diversity that exists within and between these groups. This includes distinct languages, cultural practices, kinship structures, and knowledge systems that shape health, wellbeing, and community priorities.

Additionally, the authors recognise that Indigenous women and Indigenous people with a cervix likely have distinct needs, preferences, and experiences in relation to HPV self-collection implementation. However, given the limited availability of disaggregated literature, the decision was made to combine these populations in the search strategy to ensure a more comprehensive synthesis of available evidence.

Finally, the included countries were chosen because they have experienced similar, though distinct, histories of British settler colonisation, and share comparable health system structures and policy environments that make cross-country comparisons meaningful. They also face shared structural inequities in cervical screening programs that disproportionately affect Indigenous peoples.

### Identifying relevant studies

Database searches were conducted by one author (LM) on PubMed, Scopus, and ProQuest Cumulative Index to Nursing and Allied Health Literature (CINAHL) using the broad search terms: cervical screening OR human papillomavirus, self-collection, and Indigenous peoples. These were restricted to the Title, Abstracts and Keywords. Search strings used Boolean operators to combine terms (e.g., “Indigenous OR Aboriginal OR Māori OR American Indian OR First Nations” AND “cervical screening OR Pap test” AND “self-collection OR HPV testing”). A full example of a search string is provided in Supplementary File to enhance reproducibility. Recognising the recent availability of HPV self-collection the search strategy was limited to articles published between August 2013 and November 2025 and in English. The start date of 2013 was selected to align with policy milestones such as the information to support the renewal of Australia’s National Cervical Screening Program and the emergence of HPV as a primary screening method enabling the option of self-collection. The reference lists of included studies were assessed for additional articles. Grey literature was searched to complement the peer-reviewed evidence base and identify relevant reports, policy documents, community-led initiatives, conference abstracts, and theses related to self-collection for cervical screening among Indigenous populations. The search was conducted using Google and targeted websites from Indigenous-specific organisations and government agencies. Search terms, inclusion and exclusion criteria mirrored those used in the database searches (see Supplementary File).

### Study selection

Studies were selected in accordance with a priori inclusion and exclusion criteria (Table 1) by two authors (LM and DW). Where there was disagreement between authors about inclusion, these were discussed and taken to a third author (LJW) as required. Title and abstract screening were followed by full-text review, conducted sequentially using EndNote software.

**Table 1:** Study inclusion and exclusion criteria

Parameter	Inclusion Criteria	Exclusion Criteria
Population	• Indigenous peoples eligible for cervical screening – that is, women and people with a cervix - from high-income settler colonial countries Australia (Aboriginal or Torres Strait Islander), Aotearoa New Zealand (Māori), Canada (First Nations, Inuit and Métis), the United States (American Indian, Alaskan Native, Native Hawaiian)	• Study did not focus on Indigenous peoples within high-income settler colonial countries, or did not report Indigenous-specific findings
Interest	• Explores context-specific facilitators or adaptations to implementing self-collection and recommends strategies to overcome context-specific barriers	• Focuses on general cervical screening participation, acceptability of self-collection, health disparities, cancer risk factors, or diagnostic/laboratory aspects of self-collection.
Context (historical and present)	• Primary HPV-based testing using self-collection in high-income settler-colonial contexts	• Cervical screening using method other than HPV self-collection (e.g. cytology-based screening, visual inspection with acetic acid [VIA])

### Charting the data

Data relevant to the research question was extracted by two authors (LM and DW) from the publications and organised into a spreadsheet for synthesis. Extracted variables included: citation details (author, year, publication), study aim, methods, study sample (including Indigenous population and target screening group), sample size, barriers and facilitators to self-collection, recommended strategies, and evidence of effect (e.g., screening participation rates or qualitative indicators of impact).

### Collating, summarising and reporting the results

Conventional assessment tools developed using Western knowledge systems were not used to assess methodological quality in this study, as they may devalue the diverse research methodologies employed by Indigenous communities worldwide. This decision reflects Indigenous research principles and is supported by literature that critiques the applicability of conventional appraisal tools in Indigenous contexts [22, 23]. Instead, the Health Equity Implementation Framework HEIF; [24] was used to frame the findings of the review. This framework considers determinants across three domains: societal context (sociopolitical, physical structure, economic), contextual level (local, organisational, healthcare system), and personal/clinical encounter level (recipient, provider, other recipients, innovation). Qualitative data were synthesised using thematic analysis principles, identifying patterns and themes related to barriers, facilitators, and implementation strategies. Where available, quantitative findings, such as screening participation rates, were narratively summarised to describe the reported impact of strategies. Both types of data were mapped to a matrix aligned to the HEIF framework to enable integrated analysis across domains. For each paper, data were extracted and mapped by two authors (LM, DW) and then synthesised through discussion with a third author (LJW). Thematic analysis principles were used to summarise findings into overarching themes.

## Results

**Fig. 1:**
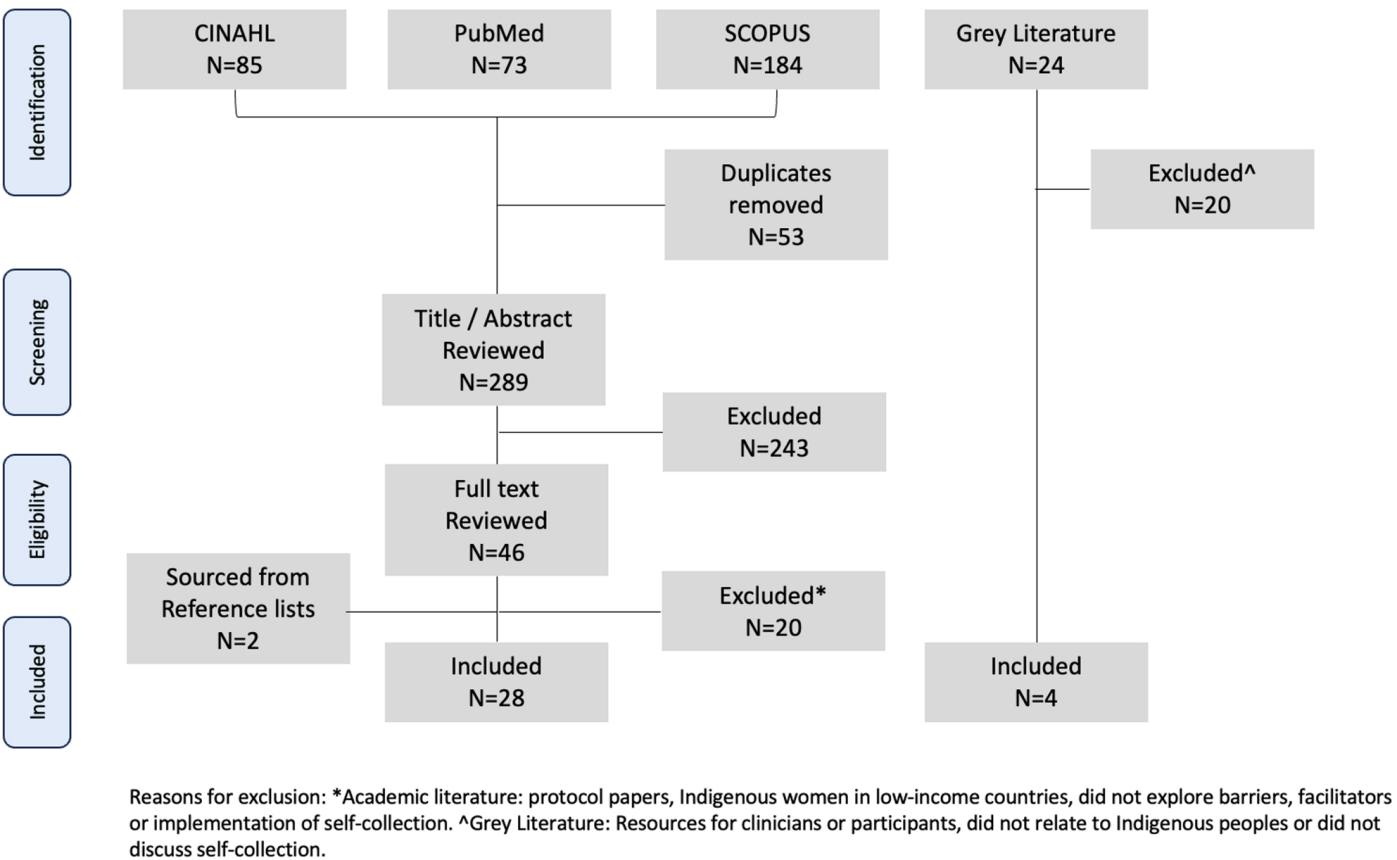
Flow chart of study selection

Searches were carried between March 2023 to November 2025 using the strategy outlined above. The process is summarised in Fig. 1. The final set of publications consisted of 32 studies, including 28 academic publications and 4 from the grey literature. Grey literature sources provided contextual insights into barriers and facilitators, helping to situate academic findings (Table 2).

**Table 2:** Included studies

Citation	Population	Study Type	Methods	Sample size	Objective
Screening Matters Study					
Butler et al., 2022 [7]	Aboriginal and Torres Strait Islander, under- and never-screened	Qualitative	Yarning	29	Describe barriers and facilitators of screening, and strategies to support screening
Whop et al., 2022 [6]	Aboriginal and Torres Strait Islander	Qualitative	Yarning	79	Understand barriers and facilitators of self-collection
Jaenke et al. 2021 [25]	Aboriginal and Torres Strait Islander	Qualitative	Interviews	13	Understand perspectives of health care providers (HCP) delivering screening, including self-collection, to Aboriginal and Torres Strait Islander women
Nurse-led community engagement model					
Dutton et al., 2020 [9]	Aboriginal and Torres Strait Islander, under- and never-screened	Mixed methods	Program Pilot and Survey	215	Test a nurse led community-based HPV self-sampling model and assess the clinical outcomes, and acceptability of the model.
Campbell et al., 2017 [26]	Aboriginal and Torres Strait Islander	Qualitative	Interviews	47	Explore feasibility and acceptability of HPV self-sampling
Marjoram et al., 2019 [27]	Aboriginal and Torres Strait Islander	Qualitative	Case study	3	Case studies of completed self-collection pilot
He Tapu Te Whare Tangata					
MacDonald et al., 2021 [13]	Māori, under- and never-screened	Quantitative	Community based, cluster RCT	Intervention = 500 Control = 431	Test HPV self-collection vs. usual care (cytology)
Adcock et al., 2019 [8]	Māori, under- and never-screened	Qualitative	Focus groups and survey with women; interviews with HCP	Focus group = 106 Surveys = 397 Interviews = 17	Explore the potential acceptability of HPV self-testing for never/under-screened women
Adcock et al., 2021 [28]	Māori, under- and never-screened	Qualitative	Interviews	28	Understand barriers and facilitators of self-collection, understand support required to facilitate clinical pathway
Cervical screening HPV self-test study					
Brewer et al., 2021 [29]	Māori, Pacific and Asian, under- and never-screened	Quantitative	Three-arm community RCT	Clinic arm = 1,574 Home arm = 1,467 Control = 512	Test self-collection models delivered in the home or clinic setting against usual cytology screening model; understand resources to achieve follow up
Sherman et al., 2022 [30]	Māori, Pacific and Asian, under- and never screened	Qualitative	Cross sectional postal survey	376	Acceptability and preferences for self-collection
Anishinaabek Cervical Cancer Screening Study					
Zehbe et al., 2016 [31]	Canadian First Nations	Quantitative	Community RCT: self-collection vs. Pap	384	Test HPV self-collection vs. usual care (cytology)
Zehbe et al., 2017 [32]	Canadian First Nations	Qualitative	Qualitative interviews	HCP = 16 Community members = 69	Understand barriers, facilitators to implementing self-collection
HPV-self sampling feasibility study in Nunavik, Northern Québec (Illiap Paanganik Qaujisarniq)					
Gamelin et al.,2022 [33]	Inuit	Qualitative	Ethnographic study	28	Understand barriers and facilitators of implementing self-collection
Gosselin et al., 2024 [34]	Inuit	Qualitative	Questionnaire on screening preference	103	Investigate factors influencing preference for cervical screening collection method after being offered screening
Santella et al., 2022 [35]	Inuit	Qualitative	Group discussions with women and semi-structured interviews with HCPs	Inuit women = 28 HCP = 20	Understand barriers and facilitators of implementing self-collection
Tratt et al., 2020 [36]	Inuit	Qualitative	Fuzzy cognitive mapping	27	Map barriers and facilitations of implementing of self-collection
Individual studies					
Bartholomew et al., 2022 [37]	Māori and Pacific, under- and never-screened	Mixed methods	Feasibility study	86	Test feasibility of telehealth model in the self-collection pathway
Bromhead et al., 2021 [38]	Māori, Pacific and Asian, under- and never-screened	Mixed methods	Feasibility study	84	Test acceptability, uptake, and cultural appropriateness of self-collection model and supported follow-up
Cina et al., 2017 [39]	American Indian, urban	Mixed methods	Survey	96	Understand HPV knowledge and attitudes before and after education session, with offer of self-collection immediately after education
Ivers et al., 2023 [40]	Aboriginal and Torres Strait Islander	Quantitative	RCT	256	Test impact of letter vs. phone/SMS cervical screening reminders for routine cervical screening (including self-collection)
Meiselbach, et al. 2023 [10]	Aboriginal and Torres Strait Islander	Mixed methods	Semi-structured interviews, demonstration project and follow up survey.	Interviews = 9 Co-design = 6 Demonstration project and survey = 37	Identify barriers and facilitators to cervical screening participation followed by a co-design workshop to support design and implementation of a self-collection demonstration project
Moxham et al., 2021 [41]	Aboriginal and Torres Strait Islander	Qualitative	Qualitative interviews	94	Understand cervical screening awareness, behaviours, knowledge, perceptions, motivators and barriers to screening, including self-collection
Paterson et al., 2024 [42]	Rural Māori	Mixed methods	Pilot and qualitative interviews	14	Understand acceptability of self-collection with same day result using point-of-care testing and immediate colposcopy at a rural community event
Powell et al., 2025 [43]	Aboriginal (Kimberley Region of Western Australia)	Mixed methods	Pilot and acceptability questionnaire	108	Evaluate the acceptability and feasibility of a community-led cervical screening model incorporating self-collection, point-of-care HPV testing, and same-day colposcopy, integrated into routine gynaecological outreach care.
Winer et al., 2016 [44]	American Indian, Hopi reservation	Mixed methods	Feasibility study	329	Recruitment within community to undertake self-collection
Evidence syntheses					
Bartholomew et al., 2021 [45]	Māori, Pacific and Asian	Qualitative	Literature review	n.a.	Recommendations for self-collection implementation in Aotearoa New Zealand
Bryant et al., 2021 [46]	Aboriginal and Torres Strait Islander, Inuit, First Nations, Métis, native people, native Canadian, Māori, and Native American	Qualitative	Rapid review	n.a.	Review strategies that increase breast, cervical or colorectal cancer screening participation, or show promise based on the factors that influence screening practices
Styffe et al., 2020 [47]	Canadian First Nations, Aboriginal, Indigenous people from Mexico, Guatemala, Inuit, Native America, Māori	Qualitative	Scoping review	n.a.	Describe literature on self-collection to inform implementation and future research
Whop et al., 2021 [4]	Aboriginal and Torres Strait Islander, Canadian First Nations, Inuit, Metis, Native Alaskans, Native Americans, Native Hawaiians, Māori	Qualitative	Data Review	n.a.	Summarise evidence on World Health Organisation cervical cancer elimination targets, including self-collection
Australian Government, 2023 [48]	Aboriginal and Torres Strait Islander	Qualitative	Literature review	n.a.	Education and Toolkit for Cervical Screening Health Practitioners
Ministry of Health, 2021 [49]	Aotearoa New Zealand, with focus on Māori	Qualitative	Public consultation	n.a.	Present findings of the Public consultation undertaken to solicit feedback on implementing HPV self-testing in Aotearoa New Zealand

### Facilitators of self-collection

Facilitators identified across the literature were grouped into key thematic categories: health literacy and knowledge, strengths-based messaging, flexible models of care, culturally responsive care, and data and systems, further described below.

#### Health literacy and knowledge

Health literacy and knowledge of self-collection and cervical screening were consistently identified as facilitators of participation. Many Indigenous participants were previously unaware of self-collection, highlighting the need for targeted awareness and education campaigns [6, 7]. Concerns about test accuracy and self-sampling ability were common, but confidence among participants and providers increased with tailored education [4, 6, 8–10, 13, 25, 28, 31–33, 35, 36, 38, 41, 46, 48]. Co-designed materials and community-level education were implemented to increase knowledge and awareness of cervical screening utilising HPV self-collection [43], and address misconceptions, including the belief that HPV testing reflects relationship fidelity [38].

#### Strengths-based messaging

Strengths-based messaging was a key strategy to support implementation. This included reinforcing the accuracy and reliability of self-collection, addressing concerns about self-sampling, and framing self-collection as a tool for promoting health and wellbeing. As noted under *Health literacy*, many participants and providers expressed greater trust in clinician-collected samples and fears of performing the test incorrectly [30, 33, 41, 47]. Messaging that reinforced the reliability of self-collection helped build confidence among participants and providers.

Self-collection was also associated with increased privacy, bodily autonomy and control, particularly among those who might not otherwise participate in screening [4, 7, 10, 29, 31, 34, 38, 41]. Among under-screened Māori, self-collection was described as empowering and having potential to reduce inequities [28]. Messaging that linked self-collection to early detection and prevention, community wellbeing, and intergenerational health was seen as effective. Some participants viewed screening as a way to model positive health behaviours for others, reinforcing its social value [4, 7, 26, 29, 32, 38, 41].

#### Flexible models of care and delivery

Self-collection supported the delivery of flexible models of care, such as telehealth, outreach, home-based screening and models mobilised by Indigenous or community health workers, overcoming practical barriers to screening such as costs, transport, childcare or work commitments [4, 8–10, 13, 25, 27, 29, 30, 32, 35, 37, 38, 41, 42, 44]. Innovative models incorporating point-of-care testing and same-day follow-up were also identified. Powell et al. [43] implemented HPV self-collection with point-of-care testing and same-day colposcopy across six remote Aboriginal communities in the Kimberley region of Western Australia. Similarly, Paterson et al. [42] offered self-collection with point-of-care testing at a rural community event for Māori. These approaches directly addressed barriers related to remoteness and follow-up, and demonstrate the feasibility of integrating self-collection into community events.

#### Culturally responsive care

Culturally responsive care was identified as vital in supporting the implementation of self-collection. This included acknowledging the impact of colonisation and recognising the resultant distrust of mainstream healthcare providers and government services [4, 7–10, 25, 28, 31, 35, 36, 38, 42, 44–49]. In many cases, culturally responsive care was facilitated through the involvement of Indigenous health workers, community leaders, and or within community-controlled settings [9, 31, 42, 43]. The use of culturally appropriate resources and education materials, including materials in local language, depictions of Indigenous peoples in instructional materials, and the inclusion of artwork and cultural references, were critical to engagement and acceptability.

Cultural safety was also enhanced through community-led approaches. For example, Powell et al. [43] described a model where cervical screening was delivered by mainstream health services in partnership with local Aboriginal healthcare providers. Elders guided participation and promotion strategies, ensuring that community voices shaped the delivery of care and that the process was culturally grounded and respectful.

#### Data and systems to support screening

Access to data was identified as a key facilitator for self-collection implementation. In particular, the ability for providers to access information at the point of care about individuals due for screening. For services, the ability to monitor and report on the screening participation and the uptake of self-collection among Indigenous peoples were viewed as essential. To support this, evidence pointed to the need for practice-level education to utilise existing functionality within systems, and the expansion of data systems to facilitate access to relevant information and strengthen reporting processes [4, 25, 37, 40].

Evidence also highlighted misinformation among providers, particularly in the context of changing clinical guidelines. To address this, provider-level education was recommended to support clear and consistent communication with clients and ensure adherence to current clinical guidelines [13].

Policy and funding mechanisms were identified as critical enablers of self-collection. Recommendations included pathology and billing models that supported flexible models of delivery, and ensuring systems were adaptable to reduce out-of-pocket costs for clients [29, 30, 45].

**Fig. 2:**
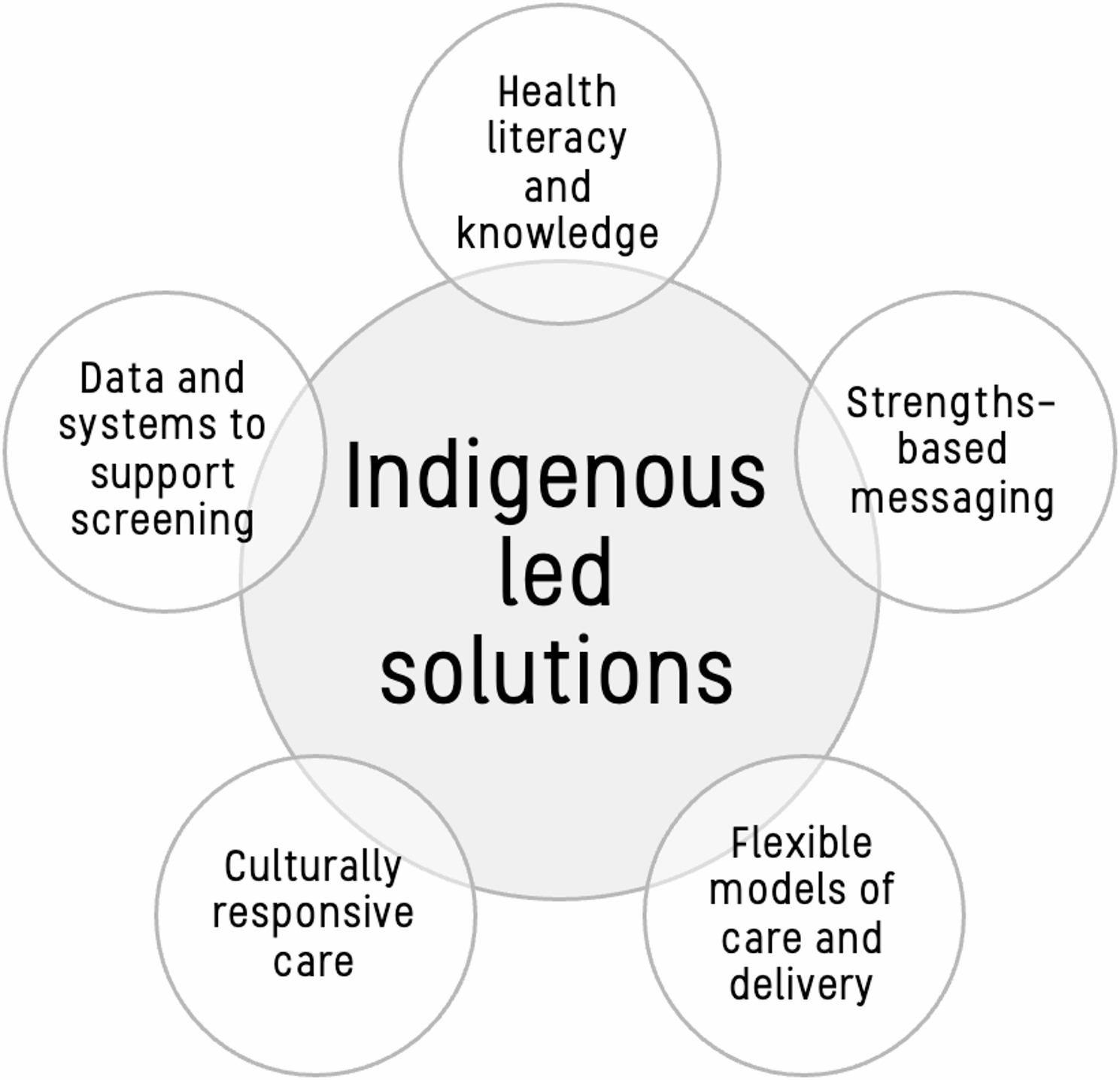
Facilitators of self-collection among Indigenous women and people with a cervix

These key themes were synthesised, with Indigenous-led solutions identified as central across all areas. A summary is provided in Fig. 2.

Recommended strategies were mapped across the HEIF (see Table 3).

**Table 3:** Strategies mapped against the health equity implementation framework (HEIF)

Domain	Strengths-based Messaging	Health Literacy and knowledge	Flexible models	Culturally Responsive Care	Data and Systems
CLINICAL ENCOUNTER	Highlight autonomy, privacy and accuracy of self-collection.	Provide time for client education and shared decision-making.	Enable self-collection during routine visits or at home; provide telehealth support when needed.	Deliver culturally competent, trauma-informed care.	Show screening status and follow-up prompts in workflows.
PERSONAL LEVEL - Community level	Promote positive messages about cervical screening and community wellbeing.	Offer community education led by trusted female healthcare providers.	Offer screening options during community activities or education sessions	Support advocacy by community leaders; build relationships based on respect and reciprocity.	Develop systems that allow individuals to access their own screening data and results easily
PERSONAL LEVEL - Client/provider level	Communicate clearly and in simple language.	Use co-designed visual aids and decision tools for client education.	Support client’s choice of collection method and location.	Develop and sustain client relationships built on trust and respect.	Train providers to use systems and clinical guidelines.
CONTEXT - Health clinic level	Encourage staff to share benefits of self-collection during education sessions.	Promote self-collection and screening through staff in-service education packages.	Provide flexible appointment models and provider options, including patient navigators, or Indigenous Liaison Officers.	Involve community members in recruitment and program co-design design.	Make screening data visible and actionable within existing clinic systems (e.g. dashboards) and simple audit tools for continuous improvement.
SOCIETAL CONTEXT	Foster positive community conversations about cervical screening	Raise awareness through social media and broad communication campaigns.	*Physical Structures*: Ensure physical structures support privacy: provide lockable spaces for self-collection and discreet packaging of kits.	*Sociopolitical forces*: Acknowledge the impact of colonisation on screening and support a culturally safe workforce, including Indigenous women’s health roles.	*Economic factors*: Advocate for interoperable systems, with real-time updates to/from registers and sustainable funding for Indigenous-led initiatives and remove out-of-pocket costs for participants.

### Impact on cervical screening participation

Thirteen studies with a quantitative component tested the impact of strategies on cervical screening participation. These included pilots of self-collection (*n* = 9) or randomised controlled trials (RCT) comparing self-collection to standard cytology (*n* = 3) or testing recall methods (*n* = 1). These are described in Table 4.

**Table 4:** Impact on cervical screening participation

Citation	Population	Methods	Strategies implemented	Outcome: Screening participation
Bartholomew et al., 2022 [37]	Māori and Pacific, under- and never-screened	Pilot	Nurse led telehealth model of self-collection at home with mailed kits	70.9% of contactable women participated in screening (31.8% of eligible women invited)
Brewer et al., 2021 [29]	Māori, Pacific and Asian, under- and never-screened	Three-arm community RCT	Two self-collection models: clinic (invited to take a self-sample at their usual general practice), home (home-based self-sampling, in which women were mailed a kit and invited to take a self-sample at home) against usual care (offered standard cytology at a clinic) with support provided to HPV positive women via phone, in person and with transport to attend colposcopy	Participation was higher for self-collection in the home (Māori odds ratio [OR] = 9.7, Pacific OR = 6.0), and for self-collection in the clinic (Māori OR = 4.1, Pacific OR = 3.3), compared to usual care
Bromhead et al., 2021 [38]	Māori, Pacific and Asian, under- and never-screened	Pilot	Co-designed patient information materials tailored to the health literacy demands placed on women and to ensure cultural appropriateness	84 of 114 (74%) contactable women completed self-collection (of 366 eligible, 23% total participation) completed self-collection
Cina et al., 2017 [39]	American Indian, urban	Pilot	HPV and cervical cancer education to urban American Indian women, followed by offer to undertake self-collection immediately following the education session	96 women participated in education. HPV knowledge exam: 61.6% pre- to 84.3% post education. 93 women performed self-collection, with 63% preferring self-collection over conventional screening methods
Dutton et al., 2020 [9]	Aboriginal and Torres Strait Islander, under- and never-screened	Pilot	Local female employee or community member recruited and supported women to screen using self-collection, with clinical support as required. All results were followed up with clients. Recruitment was at local community events, but mostly via home visits, and snow balling	Recruited 81% of target sample size to participate in screening (*n* = 215); >90% of women (*n* = 200) were highly satisfied with the HPV self-sampling kit, the service model and the process involved
Gosselin et al., 2024 [34]	Inuit	Pilot	Offer of opportunistic self-collection (either at home or in clinic) at health centre, supported by instructional materials, followed by a questionnaire to determine preference.	Of 104 eligible women, 103 agreed to participate. Twelve (11.6%) opted for nurse-collected sample, and 91 (88.3%) chose self-collection. Among these, 80.2% preferred self-sampling over the Pap test, and 82.4% would choose self-sampling in the future.
Ivers et al., 2023 [40]	Aboriginal and Torres Strait Islander	RCT	Explored the role of reminder systems, including opportunistic reminders - letter vs. phone call/SMS for cervical screening recalls. Note this was under the pre-July 2022 restricted model of self-collection - women over 30, who were at least two years overdue for screening, who had declined speculum collection, and who had no recent abnormal tests were mailed or verbally given additional information about options for self-collection.	Of 256 women, 15 (12.5%) attended screening following a letter, and 24 (17.5%) following a phone call with follow up SMS (non-significant). Proportion of self-collection not reported.
MacDonald et al., 2021 [13]	Māori, under- and never-screened	Community RCT	Staff given education and training on HPV self-collection. Self-collection offered to under- and never-screened Māori at usual primary care clinic, invited via the usual methods (text, email, letter etc.). Outreach services using nurses and health workers: health workers often reached families using home visits. All HPV types were referred to colposcopy as found preferable to Māori women.	HPV self-collection: 59.0% participation (295/500), comprising 50.8% HPV self-test (254/500) and 8.2% smear test (41/500), 2.8 times higher after adjustment for covariates. Standard care (smear test): 21.8% screened
Meiselbach, et al. 2023 [10]	Aboriginal and Torres Strait Islander	Pilot	Implementation of a demonstration project offering universal access to self-collection following co-design with community members and staff	37 women participated in demonstration project. All chose self-collection, 33% said they would not have screened without this option and 81% preferred self-collection.
Paterson et al., 2024 [42]	Rural Māori	Pilot	Outreach model (at a rural community event) incorporating self-collection, point-of-care HPV testing and colposcopy by a mobile colposcopy service	14 women screened at the event, with six testing positive for HPV and offered immediate colposcopy
Powell et al., 2025 [43]	Aboriginal (Kimberley Region of Western Australia)	Pilot	A community-led model was integrated into routine gynaecological outreach care at the invitation of participating communities. The approach included small-group education sessions using culturally appropriate materials, and incorporated self-collection, point-of-care HPV testing, and same-day mobile colposcopy.	Of 844 eligible women, 303 (36%) were invited to participate, and 108 (36% of those invited) took part within 4 months. 99% of participants recommended the approach.
Winer et al., 2016 [44]	American Indian, Hopi reservation	Pilot	Flyers and information brochures on HPV self-collection were posted in public places and distributed during tribal community events, health education campaigns and aired on the tribal radio station.	Around 10% of screen eligible women participated. Of those, 92% of enrolled participants returned a self-collected sample (329 of 353 enrolled participants). 62% reported a preference for self-collection
Zehbe et al., 2016 [31]	Canadian First Nations	Community RCT	Community health research assistants recruited participants to study. Self-collection arm: participants provided with self-collection kits and asked how they would prefer to be contacted in the event of a positive HPV test result. Engagement varied between communities based on local implementation.	Initial uptake of self-collection was 1.4x higher than cytology-based testing (20% vs. 14.3%). Range of uptake within communities varied for both self-collection (0.0% to 62.1%) and cytology (0.0% to 47.1%).

Implementation strategies included healthcare provider and community education sessions [13, 39], co-designed culturally appropriate materials [10, 38], and flexible delivery models such as home-based [9, 13, 29], telehealth supported [37], and outreach within community events [9, 42, 43]. While most studies collected participation data, few directly compared the effectiveness of different implementation strategies (see Table 4 and Supplementary Table).

Invitation methods to participate in cervical screening varied and included healthcare providers [13, 34, 42, 45], community workers [9, 31, 43] or media (e.g. emails, letters or flyers) [13, 40, 44]. One RCT compared a mailed letter with a phone call and SMS reminder. Although not statistically significant, the phone/SMS group had higher screening uptake (17.5%) than the letter group (12.5%) [40].

In a three-arm community RCT, Māori women offered home-based self-collection were 9.7 times more likely to screen than those offered standard cytology, and 4.1 times more likely than those offered clinic-based self-collection [29]. Despite these relative increases, overall participation remained low (14.6% in the home arm, 7.0% in the clinic arm, 2.0% in control). Among Canadian First Nations women, self-collection also improved uptake (1.4 times higher than control), but participation remained below 25% and varied widely across communities [31]. Community outreach models were particularly effective, with higher participation (59% compared to 22% among controls) demonstrated among under- and never-screened Māori opportunistically offered self-collection at clinics or during outreach visits by trusted primary care providers, including nurses and kaiāwhina (non-clinical community Māori health workers) [13].

Overall, self-collection consistently increased cervical screening participation among Indigenous women and people with a cervix compared to clinician-collected screening. Implemented strategies typically combined flexible delivery models, such as home-based or outreach options, with tailored education aimed at improving knowledge and building trust. Community-led initiatives and the involvement of Indigenous health workers and community leaders were central to enhancing cultural safety.

## Discussion

This scoping review mapped the literature on barriers and facilitators of HPV self-collection and identified strategies to support its implementation. Key facilitators identified included health literacy and knowledge, messaging, flexible models of care, culturally responsive care, data and systems, and Indigenous-led solutions. The use of multi-component, community led interventions were the most effective at increasing cancer screening participation in Indigenous populations. While many of the included studies reported on participation rates, few directly compared the effectiveness of different implementation strategies. Although the most effective combination of strategies remains unclear, tailored education to improve knowledge and build trust in self-collection alongside flexible delivery models (such as home-based or outreach options) were commonly used. Indigenous-led approaches and the involvement of Indigenous health workers and community leaders were central to enhancing cultural safety. Together, these findings present practical solutions to support implementation and offer a foundation for designing context-specific, culturally safe implementation strategies. However, further research is needed to identify optimal strategies for reaching under- and never-screened Indigenous women and people with a cervix whilst ensuring follow up of HPV positive results, and to explore differences across diverse Indigenous populations.

To ensure these strategies are effective and sustainable, they must be grounded in Indigenous leadership and co-design principles that reflect community values and priorities. Principles of co-design offer a pathway to achieving this shift. Anderson et al. outlined six principles for designing cervical screening interventions: First Nations leadership, culturally grounded approaches, respect, benefit to community, inclusive partnerships, and transparency and evaluation [50]. While developed in the Australian context, these principles align with global Indigenous health research frameworks, which similarly emphasise Indigenous leadership, culturally grounded care, and community-defined priorities, reinforcing the relevance and applicability of co-design principles across diverse Indigenous contexts [51–53]. These principles are essential for developing self-collection implementation strategies that align with community values and needs. This approach ensures interventions go beyond being culturally safe to becoming Indigenous led, thereby fostering ownership and sustainability.

Realising this shift requires reconfiguring governance structures to support Indigenous decision-making. This includes moving decision-making authority from traditional clinical hierarchies, such as doctor-centred models of cervical screening and colonial healthcare systems, towards Indigenous consumers, Indigenous communities, and Indigenous providers. However, systemic barriers continue to limit Indigenous leadership in health system design and delivery, despite the expertise and knowledge held within communities. Embedding Indigenous governance in program design and delivery is necessary, not only to uphold the right to self-determination but enhance uptake of self-collection [6]. Addressing these barriers through policy and system reform is essential to achieving equitable participation in cervical screening and progressing toward cervical cancer elimination.

Improving health literacy and awareness of self-collection were seen as important facilitators to support screening participation. Low health literacy, likely compounded by language barriers, limited access to culturally safe services, and stigma around sexual health and Women’s Business, consistently limits engagement with cancer screening services among Indigenous women [54–56]. This likely results in an increased likelihood of presenting with later stage disease. While participant health literacy a known determinant of screening uptake and cancer outcomes, there remains a gap in evidence about effective health literacy interventions tailored to Indigenous peoples [55, 57]. Providers play a key role in improving health literacy, through clear, effective communication. In addition, the quality and format of patient education materials play a vital role in supporting health literacy and informed screening participation. Patient education materials are widely used to support health professionals and have been found to increase cancer specific health literacy, however to date there is no research specific to Indigenous peoples [58]. A recent review of gynaecological cancer resources for Aboriginal and Torres Strait Islander women found that while there were a number of resources to support cervical cancer prevention through vaccination and screening, additional resources were required to support health literacy across the cancer care continuum [59].

Effective education and awareness resources were those that adopted co-designed principles, developed in partnership with communities, and were visually engaging, language-appropriate, and culturally relevant to ensure accessibility and resonance. Positive messaging was recommended to highlight that self-collection is accurate, supports health and wellbeing, and promotes bodily autonomy. This aligns with the strengths-based, Indigenous led approach outlined above, which emphasises the resilience and strength of Indigenous women and people with a cervix, and the positive impact of screening. Framing self-collection as a tool to promote health and community wellbeing reflects Indigenous peoples’ understanding of wellbeing. This includes autonomy, family and community, and culture, in addition to an individual’s physical health [60, 61]. Deficit-based narratives in health communication, policy and practice lead to poor health outcomes [62]. Instead, strategies should centre positive messaging. To maximise impact, these should be supported by systems that embed health literacy into service design and delivery, such as into patient pathways, staff training, and organisational policies [63].

A key advantage of self-collection identified within the literature was its flexibility, with models incorporating telehealth [37], outreach [42], home-based [13, 29], and community event-based screening utilising point-of-care testing [42, 43]. While point-of-care testing and same day colposcopy models were shown to be acceptable and feasible, further research is needed to evaluate their implementation into national screening and workforce capacity. These models have informed Australia’s National Strategy for the Elimination of Cervical Cancer [64], and have demonstrated feasibility and cost-effectiveness among women in low- to middle-income countries [65, 66]. These flexible approaches contribute to equity by reducing logistical barriers, particularly for participants in regional and remote areas. Evidence also suggests an emerging preference for home-based self-collection models [29]. This finding is consistent with evidence from other studies showing that in-person or mail-out distribution of self-collection kits is effective at increasing cervical screening participation among under-screened populations, including Indigenous women and people with a cervix [67, 68]. However, the generalisability of these findings to under- and never-screened individuals, and across diverse Indigenous populations requires further exploration given differences in health systems, policies and funding. Importantly, flexible models of care, particularly home-based or mail-out models, must align with clinical recommendations, including triaging of symptomatic participants for a clinician-collected cervical sample, and ensure timely follow-up for participants with higher risk test results [69].

While universal access to self-collection is likely to improve equity, to realise equitable elimination it must be embedded within a culturally safe system. For example, Brewer et al. [29] found that barriers to clinic attendance were similar for both self- and clinician-collected samples highlighting that self-collection along may not overcome systemic barriers to access. Mainstream institutions have a responsibility to ensure cervical screening services are culturally safe. Racism and colonisation continue to impact Indigenous peoples’ health and access to healthcare, contributing to negative mental and general health [70–72]. Racism is a major barrier towards achieving equitable health outcomes, including cervical cancer [73–75]. Improving cultural safety and responsiveness within health systems can improve access and quality care for Indigenous peoples [62, 76], with a culturally safe environment and relationship with the health provider as key [11, 77]. This requires acknowledging the impact of colonisation and addressing the resultant distrust of mainstream healthcare and government services.

All recommended strategies in this review identified cultural safety and Indigenous-led approaches as foundational to enhancing participation. While many strategies to implement self-collection apply broadly to priority populations, the need for access to culturally safe care and Indigenous-led solutions is unique to Indigenous women and people with a cervix. This reflects the ongoing impacts of colonisation, systemic racism, and mistrust of mainstream health systems - barriers that generic program approaches to date have not been able to overcome. Embedding these strategies is essential to support implementation and ensure Indigenous women and people with a cervix are reached. In many studies, Indigenous community health workers were key in delivering culturally safe care and facilitating strategy implementation. While varied, the Indigenous health and community workforce comprise culturally grounded professionals who deliver health services, promote wellbeing, and advocate for Indigenous peoples through roles that integrate clinical care, cultural knowledge, and community engagement [78]. For example, in Australia, Aboriginal and Torres Strait Islander Health Workers and Practitioners play a vital role in supporting culturally safe cervical screening, and there is strong workforce support for leading self-collection models that align with national strategies and community-led approaches [79]. To implement these strategies successfully, system level improvements, workforce development and training, and sustained funding models are needed to deliver culturally safe cervical screening and follow up services [80].

### Limitations

We acknowledge several important limitations of this review. First, while the identified overarching themes across Indigenous populations, it does not account for the diversity within and between these peoples and communities or the varied contexts in which health operates. Organised cervical screening also differ significantly across the countries included, with the implementation of a formal HPV-based screening program at different stages. Therefore, while common facilitators and possible strategies were identified, these will require tailoring to local Indigenous contexts and to local health capacities. The review also may have missed relevant studies due to language restrictions, database selection, and inclusion criteria, which could introduce bias. Additionally, much of the existing literature on HPV self-collection has focused on accuracy and feasibility rather than implementation, and disaggregated findings specific to Indigenous populations remain limited. This highlights the need for further research that explores implementation strategies in diverse contexts and evaluates their effectiveness in improving screening outcomes.

## Conclusion

Acknowledging that self-collection is both acceptable and feasible among Indigenous women and people with a cervix, this review sought to examine the barriers and facilitators of self-collection and present practical strategies to support cervical screening providers to reach Indigenous screening participants. In summary, effective implementation of HPV self-collection is supported by improving health literacy, strengths-based messaging, flexible delivery models, culturally responsive services, robust data systems, and Indigenous-led approaches. Community-driven, locally tailored, multi-component interventions were most successful in increasing screening participation, particularly focused on tailored education and flexible delivery models.

Cervical screening programs within high-income settler-colonial countries continue to face systemic inadequacies in delivering culturally safe and accessible healthcare for Indigenous women and people with a cervix. While HPV self-collection presents a promising opportunity to support equitable cervical cancer elimination, used in isolation it will not achieve this goal for Indigenous peoples. Strategic priorities have been identified to guide progress, yet their implementation depends on sustained political commitment, adequate funding, and meaningful system-level change [49, 64]. To embed these approaches sustainably, system-level changes are needed to centre Indigenous-led solutions and ensure health systems are equipped to support culturally safe, community-informed models of care. Future research should focus on implementation science approaches that evaluate the effectiveness of these strategies in diverse settings and support evidence-informed reform.

## Supplementary Information

Supplementary Material 1.

Supplementary Material 2.

Supplementary Material 3.

## Data Availability

No datasets were generated or analysed during the current study.
