# Supplementary material for: Implementing HPV self-collection: a scoping review of facilitators and strategies among Indigenous women and people with a cervix
Source: BMC Public Health. 2026 Feb 23;26:1053. doi: 10.1186/s12889-026-26720-x (PMC13037132; doi:10.1186/s12889-026-26720-x)
Supplement: Supplementary file 1 — Supplementary Material 1. [file 12889_2026_26720_MOESM1_ESM.docx]

**PubMed Search**

(cervix[Title/Abstract] OR HPV[Title/Abstract] OR vagina*[Title/Abstract] OR papillomavirus[Title/Abstract] OR cervical[Title/Abstract])

AND (self*[Title/Abstract]) AND (sampl*[Title/Abstract] OR collect*[Title/Abstract] OR screen*[Title/Abstract])

AND (Indigenous[Title/Abstract] OR aborigin*[Title/Abstract] OR maori[Title/Abstract] OR first nation*[Title/Abstract] OR inuit[Title/Abstract] OR metis[Title/Abstract] OR "Indigenous Peoples"[Mesh] OR "Health Services, Indigenous"[Mesh] OR "Australian Aboriginal and Torres Strait Islander Peoples"[Mesh] OR "Native Hawaiian or Other Pacific Islander"[Mesh] OR "Maori People"[Mesh] OR "American Indian or Alaska Native"[Mesh] )

**SCOPUS Search**

(cervix OR HPV OR vagina* OR papillomavirus OR cervical) AND (self*) AND (sampl* OR collect* OR screen*) AND (Indigenous OR aborigin* OR maori OR first nation* OR inuit OR metis)

**ProQuest CINAHL**

noft(cervix OR HPV OR vagina# OR papillomavirus OR cervical) AND noft(self# OR sampl# OR collect# OR screen#) AND noft(Indigenous OR aborigin# OR maori OR first nation# OR inuit OR meti#)

**Grey Literature**

- Google search: (cervix OR HPV OR vagina* OR papillomavirus OR cervical) AND (self*) AND (sampl* OR collect* OR screen*) AND (Indigenous OR aborigin* OR maori OR first nation* OR inuit OR metis)
- Preference for Indigenous-specific organisations:
  - Australian Indigenous HealthInfoNet
  - Lowitja Institute
  - NACCHO
  - Aboriginal Medical Services
  - AIATSIS Collection
- Government websites from CANZUS countries:
  - Australian Department of Health and Aged Care
  - Te Puni Kōkiri (NZ)
  - First Nations Health Authority (Canada)
  - U.S. Indian Health Service
  - State and territory health departments
- Conference proceedings and abstracts from relevant Indigenous health and public health events
